# Percutaneous ultrasound‐guided A1 pulley release utilizing a modified 20‐gauge spinal needle

**DOI:** 10.1002/pmrj.13276

**Published:** 2024-11-19

**Authors:** Mark Sederberg, Ragav Sharma, Daniel M. Cushman, Jonathan T. Finnoff

**Affiliations:** ^1^ Advanced Sports and Orthopaedics Lehi Utah USA; ^2^ Department of Physical Medicine & Rehabilitation University of Utah Salt Lake City Utah USA; ^3^ Department of Physical Medicine and Rehabilitation The Medical College of Wisconsin Milwaukee Wisconsin USA; ^4^ Department of Orthopaedics University of Utah Salt Lake City Utah USA; ^5^ Department of Sports Medicine US Olympic & Paralympic Committee Colorado Springs Colorado USA; ^6^ US Coalition for the Prevention of Illness and Injury in Sport Colorado Springs Colorado USA; ^7^ Department of Physical Medicine and Rehabilitation University of Colorado School of Medicine Denver Colorado USA

## Abstract

**Background:**

Trigger finger is a common cause of hand pain. Though multiple techniques for percutaneous A1 pulley release have been described in the literature, there is a continued need for safe and effective techniques using inexpensive, familiar, and commonly found instruments. This study evaluated outcomes of percutaneous A1 pulley release performed using a novel technique with a modified 20‐gauge spinal needle and ultrasound guidance, with follow‐up outcomes at least 6 months after the procedure.

**Objective:**

To evaluate the efficacy and safety of a novel percutaneous A1‐pulley release technique in individuals with trigger finger.

**Design:**

Retrospective observational study.

**Setting:**

Private practice outpatient orthopedics clinic.

**Participants:**

Forty digits from 30 unique patients with trigger finger who underwent percutaneous A1 pulley release.

**Interventions:**

Percutaneous ultrasound‐guided A1 pulley release performed with a modified 20‐gauge spinal needle.

**Main Outcome Measures:**

The primary outcome measure was cessation of triggering. Secondary measures examined intraoperative and postoperative pain, postprocedural duration of activity limiting pain, and time to perform the procedure.

**Results:**

Immediate cessation of triggering was achieved in all 40 digits following the procedure, with no recurrence reported at any time at an average follow‐up of 11 months (range 6–32). Patients reported returning to normal activity in 2.75 days. Only one minor complication was reported, tenosynovitis, which resolved with a corticosteroid injection.

**Conclusions:**

Percutaneous, ultrasound‐guided A1 pulley release performed with a modified 20‐gauge spinal needle can be safely performed with good outcomes and a rapid return to normal activity.

## INTRODUCTION

A1 pulley stenosing tenosynovitis or “trigger finger” (TF) is a relatively common condition of the hand reportedly affecting about 3% of the population.^1^ TF is named due to the catching and releasing noted in the affected fingers and most commonly affects the first (A1) pulley due to higher loads experienced by this pulley.^1^ Conservative treatment options include stretching, splinting, anti‐inflammatory medications, and tendon sheath steroid injections, with surgical treatment reserved for those recalcitrant to conservative measures.^2^ Surgical options include open and percutaneous approaches, with percutaneous approaches generally having decreased recovery time and better cosmetic results.^2^, ^3^ With the emergence of ultrasound guidance (USG), percutaneous release has become more common and an alternative to the gold‐standard open release due to lower rate of complications and decreased recovery time.^2^, ^4^

Multiple techniques to perform ultrasound‐guided A1 pulley release (USGPR) exist. A recent systematic review discussed several approaches including hypodermic needles,^5^ hook knives,^6^ aspiration “Nokor” needles (an 18‐gauge needle with a blade at the tip),^7^, ^8^ needle knives,^9^ transecting threads,^10^ and instruments designed specifically for A1 pulley release.^11^ Approaches utilizing Nokor needles or hook knives may more easily result in inadvertent damage to surrounding tissue as they continuously cut while advancing or withdrawing the needle, respectively. This may require more skill and practice prior to safe implementation, though published studies in USGPR performed by skilled providers using these instruments have shown them to be safe and efficacious.^7^, ^8^ Common techniques using plain hypodermic needles have largely used 18‐ or 20‐gauge needles but have not achieved results seen with open release due to incomplete release.^12^, ^13^ Specifically‐designed instruments and transecting threads show promise but are more costly than spinal, hypodermic, or Nokor needles. Thus, there remains a need for techniques that are safe and efficacious utilizing inexpensive, readily available tools.

In this study, we report on the safety and efficacy of a novel technique for USGPR utilizing a modified 20‐gauge spinal needle fashioned into a cutting device. To the authors' knowledge, there are no prior published studies of A1 pulley releases utilizing this technique.

## METHODS

### Study design

This was an observational retrospective study carried out at a community‐based private practice orthopedic clinic. Data were collected from medical records and from telephone interviews. Each patient was called by the physician performing the procedure or a clinic staff member 7 days after the procedure. Contact was also made either in clinic or by phone with each patient a minimum of 6 months after their procedure to check on adverse events or recurrence of triggering.

### Patients

Consecutive patients who met the following inclusion and exclusion criteria and underwent USGPR were included in the study. Inclusion criteria included presence of TF (confirmed with both physical examination and on ultrasound by a fellowship‐trained sports medicine physician) with lack of improvement following conservative care, including splinting, rest, and/or corticosteroid injection. Exclusion criteria included prior A1 pulley release in the same digit, or additional pathology in the same digit that could contribute to the triggering (eg, ganglion cyst, involvement of the A2 pulley, metacarpophalangeal joint synovitis, etc.).

### Physician and procedure

**FIGURE 1:**
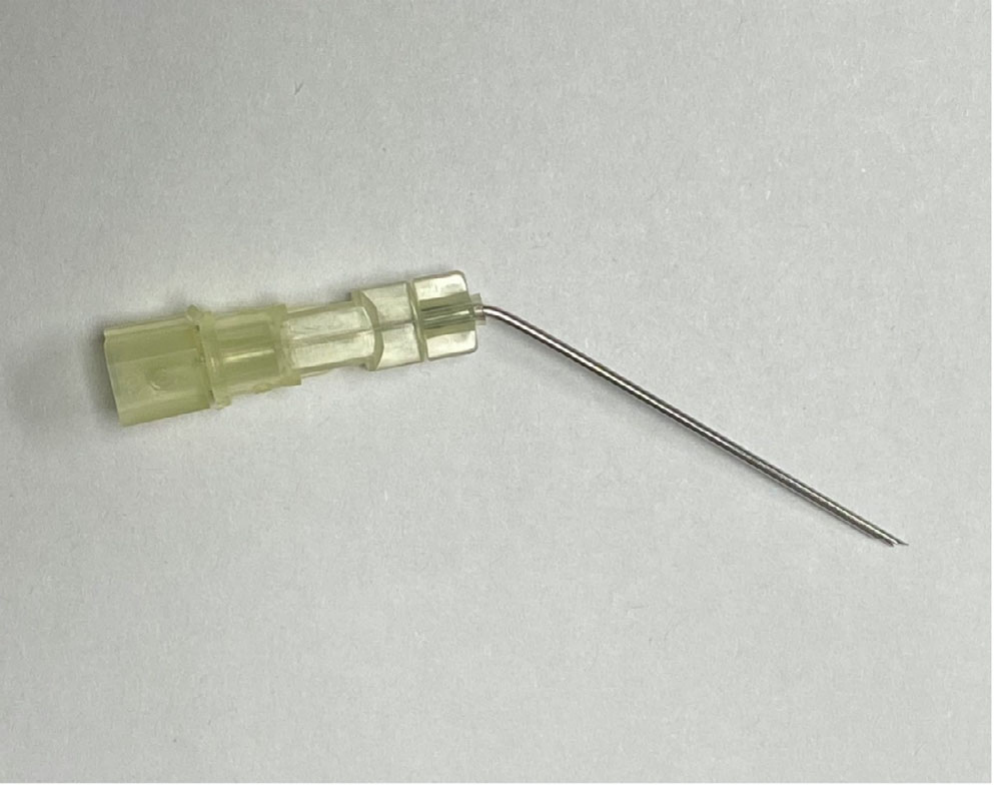
The 20‐gauge needle, with bend near the hub. The stylet is rotated 180 degrees to make a cutting blade at the tip.

**FIGURE 2:**
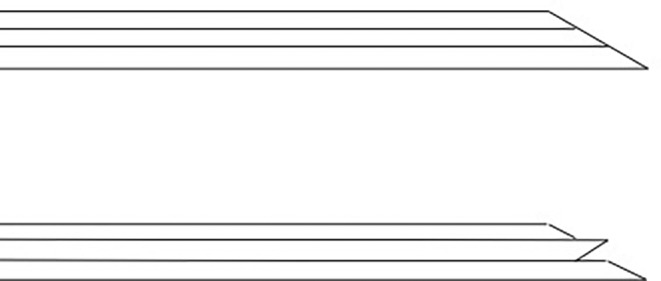
Graphic demonstrating the cutting edge made by flipping the spinal needle stylet 180 degrees.

The procedures were performed in clinic by a single sports medicine fellowship‐trained physiatrist (M.S.) with 6 years of ultrasound‐guided intervention experience. A General Electric Logiq E ultrasound machine (Wauwasota, Wisconsin) with a high‐frequency 12–8 mHz probe was used for visualization. Additional supplies used during the procedure included a 27‐gauge, 38‐mm needle for local anesthesia; 4 mL of 0.5% lidocaine without epinephrine; 10 mg of triamcinolone acetonide (40 mg/mL); and a 20‐gauge, 38‐mm spinal needle (Figure 1). This needle was bent about 60 degrees, about 6 mm from the hub. The needle hub was detached, so that the stylet was able to be rotated 180 degrees from its normal position. When the stylet was rotated 180 degrees and reinserted into the needle, the tip of the stylet protrudes out the end of the needle, creating a v‐shaped cutting tip (Figure 2).

**FIGURE 3:**
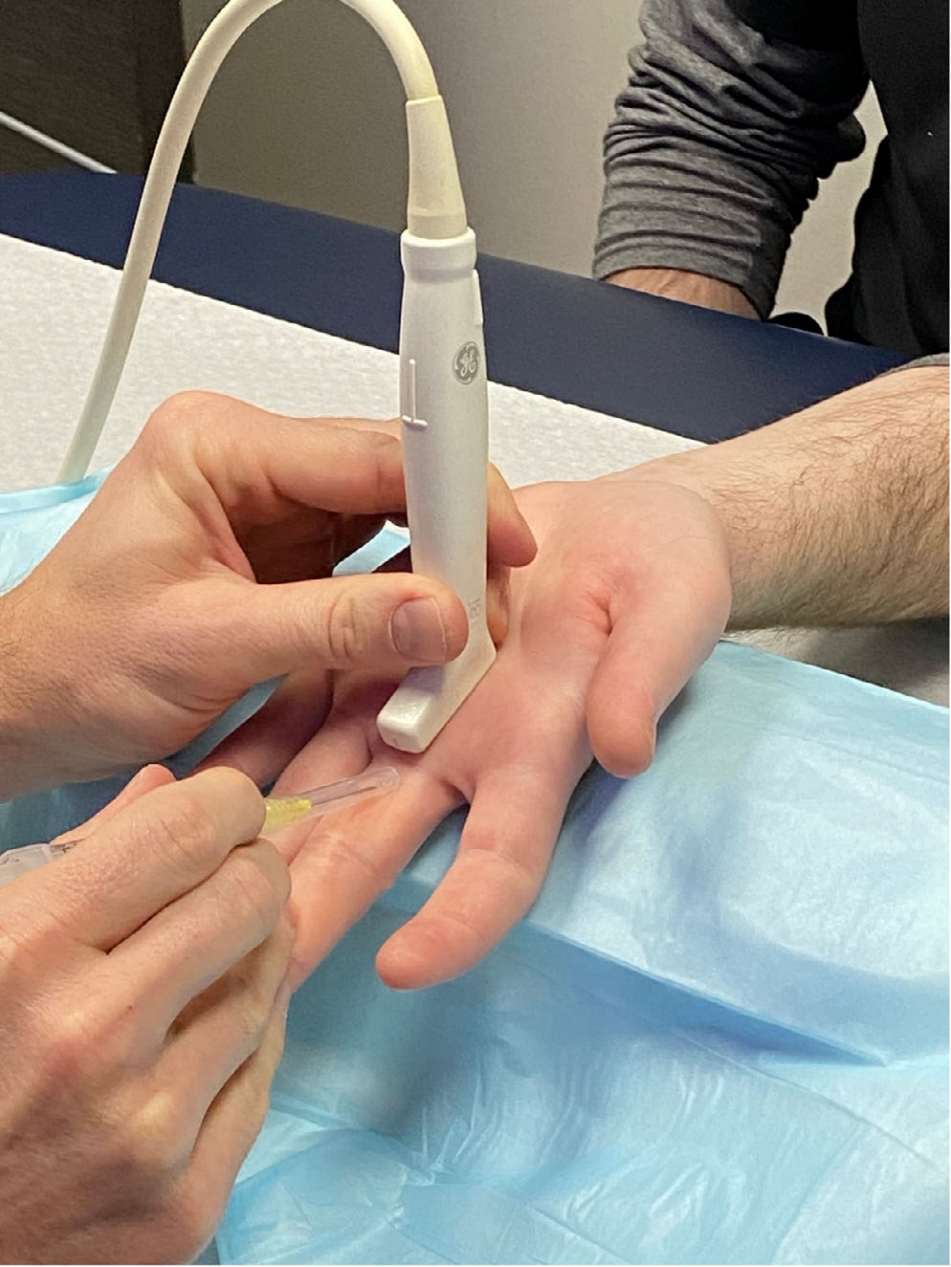
Positioning for the procedure. If the patient has limited extension at the metacarpalphalangeal joint, it is helpful to have an assistant hyperextend the metacarpalphalangeal manually.

Informed consent was obtained prior to starting the procedure. The area was cleaned with chlorhexidine and sterile ultrasound gel was utilized on the probe with a sterile probe cover. The patient was typically seated on the opposite side of the examination table from the proceduralist, and their hand was placed on the table in full supination. A rolled towel was placed under the patient's hand to allow for hyperextension of the metacarpophalangeal joint for the procedure (Figure 3). When releasing a trigger thumb, the patient was supine with the palm fully supinated at their side for easier access to the thumb A1 pulley.

**FIGURE 4:**
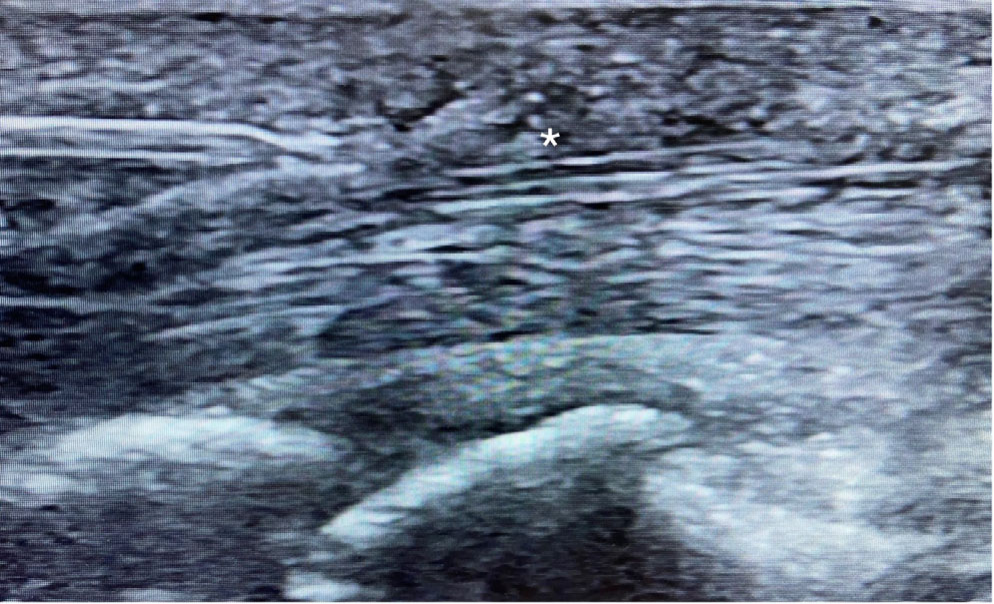
Long‐axis ultrasound image of the needle tip, entering from the left side of the screen, distal to the A1 pulley (*).

Local anesthesia was achieved with about 2.5 mL of 0.5% lidocaine, entering distal to proximal with the 27‐gauge needle, injecting the lidocaine along the needle track, and hydrodissecting the A1 pulley from the tendon and subcutaneous tissue. The needle entry point was typically near the proximal palmar digital crease. The 20‐gauge needle was then introduced distal to proximal with the stylet in the typical position. Once the needle was advanced such that the needle tip was visualized in long and short axis at the distal margin of the A1 pulley, the stylet was rotated 180 degrees, to form the v‐shaped cutting needle (Figure 4). After again verifying correct needle placement in short and long axis, the v‐shaped cutting needle was passed several times through the A1 pulley until no further tissue resistance was present when moving the needle through the A1 pulley. Then, the needle was withdrawn, and the patient was instructed to make a fist and straighten the finger fully 10 times. If there was any sensation of triggering, the procedure was repeated until the patient no longer expected triggering. Finally, a 27‐gauge, 38‐mm needle was guided to the released pulley under direct ultrasound visualization and 0.25 mL of 40 mg/mL triamcinolone acetonide was injected around the released pulley.

Following the procedure, the patient was instructed to use ice and over‐the‐counter analgesic medications (eg, acetaminophen) as needed for pain control. The patient was instructed not to the submerge the hand in water for 48 hours, to limit activity for the remainder of the day, and then resume activities as tolerated.

### Data collection

Data recorded on the day of the procedure included severity of the triggering using the Quinnell classification^14^ system (see Table 1) at the time of release and perceived intraprocedural pain on a 0 to 10 scale. A 1‐week postprocedure follow‐up visit or phone call was performed, at which time the worst postprocedural pain on a 0 to 10 scale and duration of activity limiting pain was obtained. If the patient continued to have pain that limited activity, a call was placed again in 1 week. All patients were also contacted a minimum of 6 months after the procedure, and asked if there was any recurrence of triggering or if they experienced any adverse effects from the procedure. The procedural time was also recorded for the last 10 procedures. Procedural time was defined as the amount of time it took from the preprocedural antiseptic scrub to the completion of the procedure.

**TABLE 1:** Quinnell grading system for trigger finger.^14^

Grade	Symptoms
1	No triggering, uneven finger movement and pain
2	Actively correctable triggering finger
3	Passively correctable triggering finger
4	Locked digit

Correlation between Quinell grade and postprocedural pain and activity limiting pain duration was assessed by Pearson correlation coefficient.

## RESULTS

Forty digits from 30 unique patients were included in the analysis; 17 of the digits were on females and 23 on males with participant ages ranging from 37 to 83 (mean 59) years. The triggering severity in 15 digits were classified as grade three, 21 as grade two, and 4 as grade one. No patients were classified as grade four. Nearly all digits (87.5%) were previously treated with corticosteroid injection for the same trigger finger. Procedure time for 10 consecutive procedures averaged 8.2 minutes, with a range of 5–15 minutes. One patient was excluded from the study due to the presence of active rheumatologic tenosynovitis.

Individual data from all 40 individual digits can be seen in Table 2. Intraprocedural pain, worst postoperative pain, and days to return to normal function are presented in Table 3. Patients returned to their baseline pain an average of 2.75 days after the procedure. Of 28 cases, there was only 1 recorded complication, which was a case of tenosynovitis; this resolved with a subsequent corticosteroid injection. There were no reported cases of neurovascular damage or subjective bowstringing. The last follow up was an average of 11 months after the procedure, with a range of 6–32 months. No patient had recurrence of their trigger finger.

**TABLE 2:** Outcomes for all 40 trigger fingers operated on utilizing the modified 20‐gauge needle approach.

Age (years)	Gender	Quinnell grade	Digit	Procedural pain	Days to return to normal activity	Post‐op pain	Prior CSI	Procedural time (min)
57	M	3	4	3	3	2	Y	
57	M	3	4	3	2	2	Y	
70	F	2	4	5	1	4	Y	
83	F	3	3	4	2	2	Y	
83	F	3	4	4	2	3	N	
43	F	2	1	5	7	4	Y	
65	F	3	1	1	2	5	Y	
48	F	2	1	2	1	4	Y	
37	M	3	1	4	3	3	Y	
55	M	3	1	3	4	4	Y	
55	M	2	4	3	2	2	Y	
67	F	3	3	3	3	3	Y	
66	M	2	2	3	2	3	Y	
67	M	2	5	3	1	3	Y	
67	M	2	5	3	1	3	Y	
61	F	2	1	2	3	4	N	
70	M	2	4	6	8	6	Y	15
69	M	2	4	1	1	3	Y	8
58	M	2	1	3	3	4	Y	12
49	F	3	3	4	1	3	N	13
71	F	3	1	2	3	3	Y	6
54	M	2	2	1	4	5	Y	6
64	M	1	4	3	3	1	Y	10
45	F	2	4	2	5	4	Y	5
45	F	2	4	3	5	4	Y	5
61	M	2	2	7	3	5	Y	7
68	M	1	3	1	2	3	Y	6
68	M	1	3	1	2	3	Y	5
42	F	1	3	2	1	3	N	
62	M	3	4	4	2	3	N	
55	M	2	2	4	3	2	Y	
40	F	2	1	3	2	3	Y	
30	M	3	1	3	2	2	Y	
81	F	2	2	2	3	2	Y	
81	F	2	2	2	3	2	Y	
48	M	3	2	1	1	4	Y	
48	M	3	3	1	1	4	Y	
64	F	2	5	5	3	4	Y	
70	M	2	4	3	4	3	Y	
55	M	3	2	3	2	4	Y	

Abbreviation: CSI, corticosteroid injection.

**TABLE 3:** Median, interquartile range (IQR), and minimum and maximum values for main outcome measures.

	Median	IQR	Min	Max
Intraprocedural pain	3	2	1	7
Days to return to normal	3	1	1	8
Postoperative pain	3	3	1	8

There was no statistically significant relationship between Quinell grade and peak postoperative pain (r = .0377, *p* = .817) or duration of activity limiting pain (r = −0.107, *p* = .511).

## DISCUSSION

To our knowledge, this is the first study describing the results of ultrasound‐guided A1 pulley releases performed using a modified spinal needle to create a v‐shaped cutting tip. USGPR described in this study was safe, efficacious, minimally painful, and allowed a rapid return to normal activity. There have been several published studies analyzing the efficacy of percutaneous, ultrasound‐guided A1 pulley releases using various specialized instruments, most of which have been shown to be efficacious and safe. However, the specialized equipment increases the expense of the procedure, requires acquisition of the specialized equipment, and may lead to additional complications or delayed recovery due to more tissue trauma from a larger cutting tip that is permanently in place (eg, hook knife or Nokor needle).

Our success rate was 100%, which is similar to published findings of ultrasound‐guided A1 pulley releases using other techniques. A recent systematic review of USGPRs shows an overall success rate of 97% with 749 pooled cases.^2^ Another systematic review and meta‐analysis comparing USGPR to open surgical release of the A1 pulley demonstrated comparable efficacy and quicker improvement in hand function.^15^

There are potential advantages to this technique over those using other devices as this device is a modified spinal needle, and the cutting motion is performed as an advancement of the needle through the A1 pulley under ultrasound‐guidance. This technique may use more familiar motions to interventionalists that are already comfortable with ultrasound‐guided injections, as it is a similar motion and needle steering to a standard injection, so may feel less foreign than techniques that use other instruments such as a hook knife or surgical thread. The size of the needle is also smaller than other reported needles, which offers a more precise cutting size. The advantage of having only the cutting tip active when the needle tip is adjacent to the A1 pulley is also beneficial because it decreases the possibility of inadvertent tissue damage when introducing and removing the needle, as is potentially more possible with other instruments such as a Nokor needle or hook knife or larger gauge commercially available instruments such as the UltraGuideTFR or Sono‐Instrument. We note that even with the increased possibility of inadvertent tissue damage, reported injury is similarly low in these instruments.^8^, ^11^, ^16^, ^17^ Additionally, the cost of a single spinal needle is lower than the cost for specialized instruments and more similar to a Nokor needle or 18‐gauge needle.

Our reported days to return to normal activity is low, at 2.75 days. This included several patients with manually intensive jobs such as a plumber and landscaper, who returned to work the following day. This quick recovery is in line with other percutaneous trigger finger release studies that reported similar outcomes, ranging from 1 day to 6.6.^15^, ^16^, ^17^, ^18^ A systematic review found a mean difference of 13.8 days in return to normal activities between USGPR and open surgical release. There was no significant relationship between days to return to normal activity and Quinell grade.

Our singular complication, tenosynovitis, resolved with one tendon sheath corticosteroid injection. This complication is in line with findings in other studies.^2^ No neurovascular injury, damage to the tendon, or bowstringing was reported. In addition, no recurrence of triggering occurred on any patients. Injected corticosteroid was used after the procedure, which performed in isolation can stop triggering temporarily or permanently.^19^ However, the absence of triggering during the procedure prior to the injection of steroid indicates that the resolution of triggering was due to the USGPR rather than the steroid injection. In addition, most patients in this study had previously received a corticosteroid injection that did not provide sustained relief from triggering, making it unlikely that the corticosteroid was responsible for the long‐term cessation of triggering.

This study has several limitations. A larger sample size would give us more data as to the true efficacy of this procedure, along with improved evaluation of adverse effects. This study was retrospective, and there was no randomization nor control to compare to open surgery. Another study determined the cost of non‐image‐guided percutaneous A1 pulley release to be lower than that of open surgery.^20^ This would likely be the case in our technique, given the relatively inexpensive materials and its being performed in the office setting; however, this was not examined in our study. There is also no comparison of outcomes between races, genders, ethnicities, vocations, duration of symptoms, thumb versus other finger. The length of the procedure, 8.2 minutes, was measured on only 10 patients. This is slightly faster than the other two studies examining USGPR procedure time, which reported 15 and 9 minutes.^9^, ^21^ With familiarity of the procedure, this reported time is likely shorter than duration of earlier procedures. This small sample size is not large enough to demonstrate a difference in procedural time between thumb (which is anecdotally more technically challenging) and other digits.

It is unclear if the post‐procedural small‐dose corticosteroid injection conveyed value or is unnecessary. One randomized controlled trial found that simultaneous corticosteroid injection with non‐imaged‐guided percutaneous A1 pulley release improved subjective pain at 3 weeks, though visual analog scale (VAS) was higher at 3 months compared to release without corticosteroid injection. However, two trials comparing USGPR with and without corticosteroid injection found significantly improved VAS at 1 day and 1 month in the steroid group, and equal VAS at 1 year.^23^, ^24^ As one of the major advantages of this technique versus open technique is improved short‐term return to pain‐free activity, the steroid likely conveys additional value to most patients. There is potential concern of increased infection as a complication from adding corticosteroids, though none were noted in our patient cohort. Larger numbers would be needed to determine the risk of concurrent steroid injection and infection.

## CONCLUSION

The USGPR technique described in this retrospective case series appears safe, efficacious, and quick to complete; involves minimal procedural pain; and allows patients to rapidly return to normal activity. Furthermore, all the equipment needed to perform the procedure is readily found in the office of physicians who perform ultrasound‐guided procedures. Future studies with improved methodology are required to confirm and expand upon our preliminary findings.

## DISCLOSURES

None.
